# Assessment of contribution of BCRP to intestinal absorption of various drugs using portal‐systemic blood concentration difference model in mice

**DOI:** 10.1002/prp2.544

**Published:** 2020-01-17

**Authors:** Iichiro Kawahara, Satoyo Nishikawa, Akira Yamamoto, Yusuke Kono, Takuya Fujita

**Affiliations:** ^1^ Department of Biopharmaceutics Kyoto Pharmaceutical University Kyoto Japan; ^2^ Graduate School of Pharmaceutical Sciences Ritsumeikan University Shiga Japan; ^3^ Present address: Japan Tabacco, Inc Osaka Japan; ^4^ Present address: Shionogi & Co. Ltd. Osaka Japan

**Keywords:** breast cancer resistant protein, in vitro‐in vivo correlation, intestinal absorption, portal‐systemic blood concentration difference method

## Abstract

Prediction of the intestinal absorption of new chemical entities (NCEs) is still difficult, in part because drug efflux transporters, including breast cancer resistance protein (BCRP) and P‐glycoprotein (P‐gp), restrict their intestinal permeability. We have demonstrated that the absorptive quotient (AQ) obtained from the in vitro Caco‐2 permeability study would be a valuable parameter for estimating the impact of BCRP on the intestinal absorption of drugs. In this study, in order to assess the correlation between the in vitro AQ for BCRP and in vivo contribution of BCRP on drug absorption, we evaluated the oral absorption of various compounds by portal‐systemic blood concentration (P‐S) difference method in wild‐type (WT), Bcrp(−/−), and Mdr1a/1b(−/−) mice. In addition, we also calculated a rate of BCRP contribution (*R_bcrp_*). Ciprofloxacin and nitrofurantoin showed the low *R_bcrp_* value (0.05 and 0.15), and their apparent fractions of intestinal absorption in WT mice were 46.5% and 63.7%, respectively. These results suggest that BCRP hardly affects their intestinal absorption in mice. On the other hand, the apparent fraction of intestinal absorption of topotecan and sulfasalazine was significantly lower in WT mice than in Bcrp(−/−) mice. Moreover, their *R_bcrp_* values were 0.42 and 0.79, respectively, indicating the high contribution of BCRP to their oral absorption. Furthermore, in vivo *R_bcrp_* calculated in this study was almost comparable to in vitro AQ obtained from Caco‐2 permeability study. This study provides useful concepts in assessing the contribution of BCRP on intestinal absorption in drug discovery and development process.

**Abbreviations**
AQ: absorptive quotient
AUC: area under the plasma concentration‐time curve
AUMC: area under the first moment curve
Bcrp: breast cancer resistance protein
CLtot: total body clearance
*F*a*F*g: the apparent local absorption ratio from the gastrointestinal tract into the portal system
MAT: mean absorption time
MRT: mean residence time
P‐gp: P‐glycoprotein
P‐S: portal‐systemic blood concentration
*R*: rate of contribution
Vdss: distribution volume at the steady state

## 1 INTRODUCTION

Oral drug administration has been most frequently used in clinical because it has several advantages against other administration routes, such as easy to use, high safety, good patient compliance, and low cost. Therefore, in the development of new drug, it is very important to make many new chemical entities (NCEs) to be an orally available dosage form. However, most of the NCEs, which have been discovered recently, tend to have disadvantageous characteristics for oral administration, that is, poor water solubility, low membrane permeability, and substrate for various efflux drug transporters. In particular, at the early drug discovery stage, it is important to estimate whether each NCE is recognized by drug efflux transporter and its intestinal permeability is restricted.

In drug efflux transporters, breast cancer resistance protein (BCRP; ABCG2) expression level in human intestine has been reported to be equal to or even higher than that of MDR1.^1^, ^2^ BCRP has one adenosine 5'‐triphosphate (ATP)‐binding cassette and six transmembrane domains and is, therefore, so called a half‐ABC transporter, which forms homodimers to obtain functional activity.^3^, ^4^ Since Bcrp(−/−) mice were developed by Schinkel et al,^5^ a lot of in vivo studies using Bcrp(−/−) mice have been carried out to evaluate the effect of BCRP on the oral absorption of drugs.^6^, ^7^, ^8^ In most of these reports, systemic plasma concentration of drugs after oral administration was compared between Bcrp(−/−) mice and wild‐type (WT) mice. In case of BCRP substrate drug, its bioavailability (BA) in Bcrp(−/−) mice is tended to be higher than that in WT mice, because Bcrp is highly expressed in liver and kidney, relatively high expressed in small intestine.^6^, ^7^, ^8^, ^9^

We have evaluated the Caco‐2 permeability of various BCRP and/or P‐glycoprotein (P‐gp) substrates and defined an absorptive quotient (AQ) for estimating the specific contribution of BCRP to intestinal permeability of drugs. This in vitro assay system using Caco‐2 cells for calculating AQ might be an efficient approach to estimate the oral absorption of NCEs, particularly with respect to the contribution of BCRP. In order to demonstrate this expectation, it is required to investigate whether the estimated contribution of BCRP to intestinal permeability from in vitro study correlates with the in vivo study.

**Figure 1:**
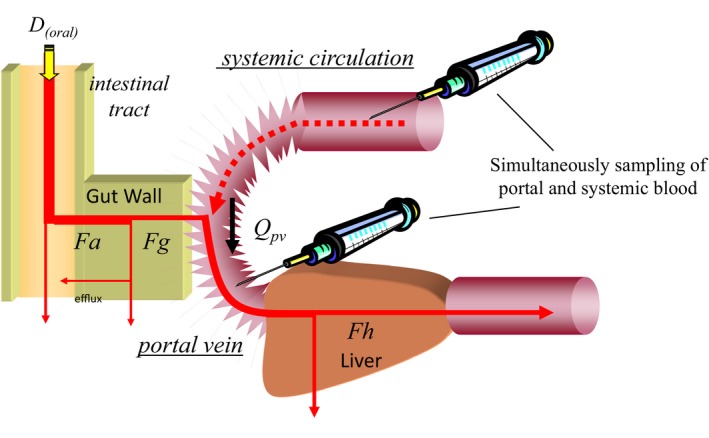
Schematic representation of P‐S difference method model

In this study, we evaluated the contribution of BCRP, as well as P‐gp, which is a representative drug efflux transporter, to intestinal drug absorption using a recirculatory model for portal‐systemic blood concentration (P‐S) difference method (Figure 1) in Bcrp(−/−) and Mdr1a/1b(−/−) mice.^10^, ^11^ This method was developed to separately evaluate the rate and extent of absorption from the gastrointestinal tract into the portal system and disposition of a drug in the body. We here applied this method for various model compounds, and estimated the apparent local absorption ratio from the gastrointestinal tract into the portal system (*F*_a_ *F*_g_) in WT, Bcrp(−/−), and Mdr1a/1b(−/−) mice. Then, we calculated the in vivo AQ values for BCRP and P‐gp, and ratios of contribution (R), which indicate the contribution of BCRP and P‐gp on the intestinal absorption. Furthermore, we also assessed the correlation of in vivo AQ with in vitro AQ obtained from in vitro Caco‐2 permeability studies.

## 2 MATERIALS AND METHODS

### 2.1 Chemical and reagents

Caffeine was purchased from Nacalai Tesque (Kyoto, Japan). Ciprofloxacin was purchased from LKT Laboratories, Inc (St. Paul, MN). Nitrofurantoin was purchased from MP Biomedicals, Inc (Tokyo, Japan). Topotecan HCl was purchased from ALEXIS CORPORATION (Lausen, Switzerland). Sulfasalazine was obtained from Sigma‐Aldrich (St. Louis, MO). Transwell^®^ was purchased from Corning (Corning, NY). Other chemicals used were of the highest purity available.

### 2.2 Animals

Male Mdr1a/1b(−/−) and Bcrp(−/−) mice, and WT mice of the same genetic background (FVB) were obtained from Taconic Farms (Germantown, NY, USA). The mice in the present study were 10 to 18 weeks old and weighed 23 to 35 g. Animals were maintained under standard conditions with a 12 hours light/dark cycle. Food and water were available ad libitum. All experiments were carried out in accordance with the principles and procedures outlined in the National Institutes of Health Guide for the Care and Use of Laboratory. All animal experimental protocols were reviewed and approved by the Animal Care and Use Committee of Kyoto Pharmaceutical University (2005‐239) and Ritsumeikan University (BKC2010‐27).

### 2.3 Preparation of drug solution

For intravenous administration studies, each of the following model compounds was dissolved in saline containing 1% dimethyl sulfoxide and 10% polyethylene glycol 400: ciprofloxacin, 0.2 mg/mL; topotecan, 0.2 mg/mL; nitrofurantoin, 0.1 mg/mL; and sulfasalazine, 0.1 mg/mL. For oral administration studies, each of the following model drug was dissolved in water with 1% dimethyl sulfoxide and 10% Solutol HS15: ciprofloxacin, 0.2 mg/mL; topotecan, 0.2 mg/mL; nitrofurantoin, 0.5 mg/mL; and sulfasalazine, 0.5 mg/mL.

### 2.4 Pharmacokinetic studies

All the mice were fasted overnight with free access to tap water. In the intravenous administration studies, model compounds were administered via the tail vein at doses of 1 mg/kg (n = 3). Following administration, blood samples were collected from the abdominal vein of the anesthetized mice at 0.083, 0.17, 0.5, 1, 2, 4, and 8 hours. In the oral administration study, ciprofloxacin, nitrofurantoin, topotecan, and sulfasalazine were administered by gavage at a dose of 1, 2, 1, and 5 mg/kg, respectively (n = 2). Following administration, blood samples were taken from the portal and abdominal veins of the anesthetized mice at 0.083, 0.17, 0.5, 1, 2, 4, and 8 hours. The plasma samples were separated by centrifugation at 14 000g for 10 minutes at 4°C and stored at −30°C until analysis.

### 2.5 Determination of blood/plasma concentration ratio (*R*_b_)

The model compounds were spiked into fresh whole blood collected from FVB mice at final concentrations of 1 µg/mL. After the incubation at 37°C for 15 minutes, the plasma samples were obtained by centrifugation at 14 000*g* for 10 minutes at 4°C. Similarly, the model compounds were added to plasma, and reference blood samples were obtained according to the same procedure. These concentrations of drugs in each sample were analyzed using HPLC (*C*_B_ and *C*_P_, respectively). *R*_b_ value was calculated by dividing *C*_B_ by *C*_P_.

### 2.6 Analytical methods

Ciprofloxacin and nitrofurantoin were extracted from the plasma with dichloromethane and ethyl acetate, respectively. After organic layer was evaporated at 60°C, the resultant residues were dissolved in a mobile phase. For the determination of topotecan and sulfasalazine, plasma samples were mixed with acetonitrile, centrifuged at 750 g for 10 minutes at 4°C, and the supernatants were collected. After the evaporation of the supernatants, the residues were dissolved in a mobile phase, and acidified with phosphoric acid for topotecan. All drugs were analyzed by HPLC system (Shimadzu LC‐10AS pump, Shimadzu SIL‐10A autosampler) equipped with a reverse‐phase column (COSMOSIL 5C_18_‐AR‐II, 3.5‐μm inner diameter, 4.6 × 150 mm). The flow rate was 1.0 mL/min. The compositions of mobile phases were as follows: ciprofloxacin, 10 mmol/L formate buffer (pH 3.0) with methanol and acetonitrile (82:9:9, v/v); nitrofurantoin, 10 mmol/L phosphate buffer (pH 3.0) with acetonitrile (83:17, v/v); topotecan, 10 mmol/L phosphate buffer (pH 3.7) with methanol (76:24, v/v); and sulfasalazine, 5 mmol/L phosphate buffer (pH 6.0) with acetonitrile (78:22, v/v). Nitrofurantoin and sulfasalazine were detected by absorbance at 366 nm and 357 nm, respectively, using Shimadzu SPD‐20A UV spectrophotometric detector. Ciprofloxacin was analysed by measuring the fluorescent intensity at a wavelength of 280 (excitation)/460 (emission) nm using Shimadzu RF‐10A XL fluorescence detector. Topotecan was also detected by measuring the fluorescent intensity at a wavelength of 361 (excitation)/527 (emission) nm.

### 2.7 Pharmacokinetic analysis

Elimination rate constant (*k*_e_) was determined by the least squares regression analysis of plasma concentration vs time curve. Elimination half‐life (*t*_1/2_) was calculated using Eq.1:

(1) $$t_{1/2}=\ln2/k_{e}$$

Area under plasma concentration‐time curve (AUC) and area under the first moment curve (AUMC) from time 0 to infinity were calculated by trapezoidal rule. Mean residence time (MRT), mean absorption time (MAT), total body clearance (CL_tot_), and distribution volume at the steady state (*V*_dss_) were calculated using following equations:

(2) $${\text{CL}}_{\text{tot}}=\text{Dose}/\text{AUC}$$

(3) $$V_{\text{dss}}={\text{AUMC}}_{\mathrm{iv}}/{\text{AUC}}_{\mathrm{iv}}\times {\text{CL}}_{\text{tot}}$$

(4) $${\text{CL}}_{\text{tot}}=\text{Dose}/\text{AUC}$$

(5) $$V_{\text{dss}}={\text{AUMC}}_{\mathrm{iv}}/{\text{AUC}}_{\mathrm{iv}}\times {\text{CL}}_{\text{tot}}$$

where AUMC*_iv_* and AUC_i_ *_v_* mean AUMC and AUC after intravenous administration, respectively.

Absorption rate constant (*k*_a_) after oral administration was calculated by the nonlinear least squares fitting with program MULTI.^12^

Apparent *F*_a_ *F*_g_ (*F*_a_, absorption ratio; *F*_g_, intestinal availability) in P‐S difference model was calculated by Eq.4:

(6) $$F_{a}F_{g}=Q_{\text{pv}}\times R_{b}\times \left({\text{AUC}}_{\text{pv}}-{\text{AUC}}_{\text{sys}}\right)/\text{Dose}\;$$

where *Q*_pv_ is the portal blood flow (106.6 mL/min/kg,^13^, ^14^ AUC_pv_ is the AUC in portal vein, and AUC_sys_ is the AUC in systemic circulation). BA was calculated by Eq.5:

(7) $$\text{BA}={\text{AUC}}_{\text{oral}}/{\text{AUC}}_{\mathrm{iv}}\times {\text{Dose}}_{\mathrm{iv}}/{\text{Dose}}_{\text{oral}}\times 100$$

where AUC_oral_ is AUC after oral administration. Dose*_iv_* and Dose_oral_ are administered dose in the intravenous and oral administration study, respectively.

Hepatic availability (*F*_h_) was calculated by Eq.6:

(8) $$F_{h}=F/\left(F_{a}\times F_{g}\right)$$

**Figure 2:**
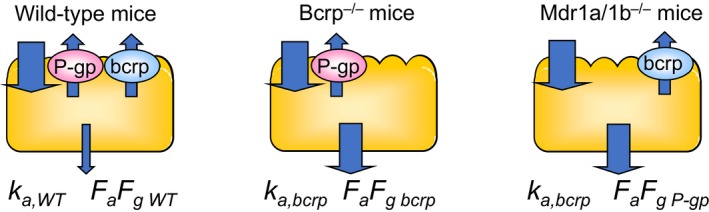
Schematic representation of in vivo measures of BCRP or P‐gp modulated drug absorption used in this study

In vivo *AQ* was defined by the following equation using *k*_a_ in WT, Bcrp(−/−), and Mdr1a/1b(−/−) (*k*_a,WT_, *k*_a,BCRP_, *k*_a,P‐gp_) (Figure 2):

(9) $${\text{AQ}}_{\text{Bcrp}}=\frac{k_{a,\text{Bcrp}}-k_{a,\text{WT}}}{k_{a,\text{WT}}+\left(k_{a,\text{Bcrp}}-k_{a,\text{WT}}\right)+\left(k_{a,P-\text{gp}}-k_{a,\text{WT}}\right)}$$

(10) $${\text{AQ}}_{P-\text{gp}}=\frac{k_{a,P-\text{gp}}-k_{a,\text{WT}}}{k_{\text{a,WT}}+\left(k_{\text{a,Bcrp}}-k_{\text{a,WT}}\right)+\left(k_{\text{a,P}-\text{gp}}-k_{\text{a,WT}}\right)}$$

In addition, we defined a rate of contribution (*R*), which indicates the contribution of P‐gp or BCRP on the intestinal absorption, by the following equation:

(11) $$R_{\text{Bcrp}}=\frac{F_{a}F_{\text{gBcrp}}-F_{a}F_{\text{gWT}}}{F_{a}F_{\text{gWT}}+\left(F_{a}F_{\text{gBcrp}}-F_{a}F_{\text{gWT}}\right)+\left(F_{a}F_{\text{gP}\;-\;\text{gp}}-F_{a}F_{\text{gWT}}\right)}$$

(12) $$R_{P\;-\;\text{gp}}=\frac{F_{a}F_{\text{gP}\;-\;\text{gp}}-F_{a}F_{\text{gWT}}}{F_{a}F_{\text{gWT}}+\left(F_{a}F_{\text{gbcrp}}-F_{a}F_{\text{gWT}}\right)+\left(F_{a}F_{\text{gP}\;-\;\text{gp}}-F_{a}F_{\text{gWT}}\right)}$$

where *F*_a_ *F*_gWT_, *F*_a_ *F*_gBcrp_, and *F*_a_ *F*_gP‐gp_ are *F*_a_ *F*_s_ in WT, Bcrp(−/−), and Mdr1a/1b(−/−) mice, respectively.

## 3 RESULTS

### 3.1 Assessment of the contribution of BCRP on the oral absorption of model drugs

We evaluated the contribution of BCRP and P‐gp to the intestinal absorption of model drugs, ciprofloxacin, nitrofurantoin, topotecan, and sulfasalazine, by P‐S difference method in WT, Bcrp(−/−), and Mdr1a/1b(−/−) mice. Prior to in vivo absorption studies, we comparatively evaluated the expression characteristics of efflux transporter, aside from BCRP and P‐gp, and drug‐metabolizing and conjugating enzymes, which are involved in drug absorption and metabolism, in mice. The mRNA expression levels of *Mrp2* in the intestine and *Cyp3a11*, *Slut1a1*, and *Ugt1a1* in the intestine and liver were not significantly different among WT, Bcrp(−/−), and Mdr1a/1b(−/−) mice (data not shown). In addition, we also determined *R*_b_ value of model drugs. The measured *R*_b_ values of all the model drugs were approximately 1.0 (ciprofloxacin, 1.20 ± 0.10; nitrofurantoin, 1.18 ± 0.10; topotecan, 0.94 ± 0.05; and sulfasalazine, 1.28 ± 0.03). These results indicate that the distribution of these drugs in plasma is almost equal to that in blood cells. Moreover, there were no differences in *R*_b_ values among WT, Bcrp(−/−), and Mdr1a/1b(−/−) mice (data not shown).

#### 3.1.1 Ciprofloxacin

**Figure 3:**
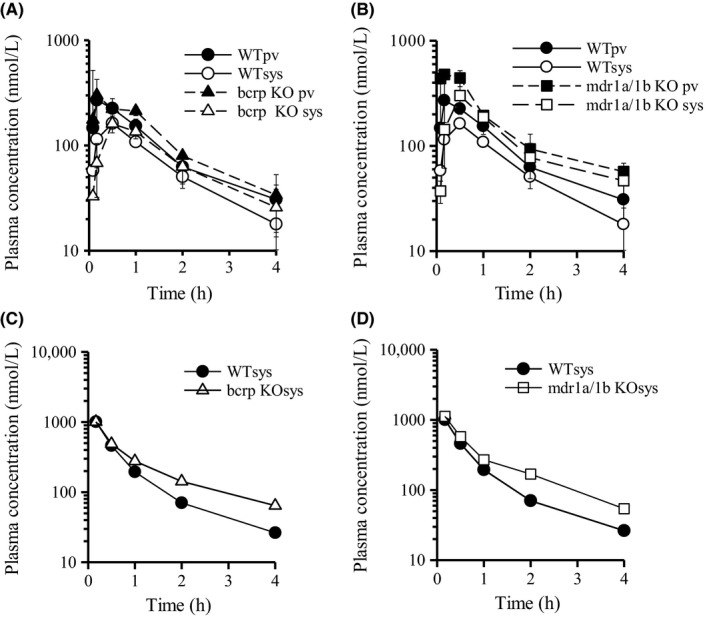
Plasma concentration vs time profiles of ciprofloxacin in WT, Bcrp(−/−), and Mdr1a/1b(−/−) mice after oral and intravenous administration. The plasma concentration vs time profiles of ciprofloxacin in WT, Bcrp(−/−), and Mdr1a/1b(−/−) mice after oral administration (1 mg/kg; A, B) and intravenous administration (1 mg/kg; C, D). Each point is expressed as mean ± SD (po: n = 3) or means (iv; n = 2)

We also evaluated the plasma concentration of ciprofloxacin following intravenous and oral administration in WT, Bcrp(−/−), and Mdr1a/1b(−/−) mice (Figure 3, Table 1). The portal plasma concentration of ciprofloxacin reached the peak at 10 minutes after oral administration in each WT, Bcrp(−/−), and Mdr1a/1b(−/−) mice. These profiles show that ciprofloxacin is rapidly absorbed from the upper small intestine. Moreover, *AUC*_pv_ and *AUC*_sys_ of ciprofloxacin after oral administration in Bcrp(−/−) mice were almost the same as those in WT mice, and the calculated *F*_a_ *F*_g_ was also nearly equal in both mice (50.6% in Bcrp(−/−) mice and 46.5% in WT mice). In addition, there was no difference in *ka* values between Bcrp(−/−) mice and WT mice (1.85 per hour and 1.63 per hour, respectively). These results indicate that BCRP hardly affects the intestinal absorption of ciprofloxacin.

**Table 1:** Pharmacokinetic parameters of ciprofloxacin after oral and intravenous administration to wild‐type, Bcrp(−/−), and Mdr1a/1b(−/−) mice

	wild‐type				Bcrp(−/−)				Mdr1a/1b(−/−)			
	iv	p.o.			iv	p.o.			iv	p.o.		
		pv		sys		pv		sys		pv		sys
Dose (mg/kg)	1.0		1.0		1.0		1.0		1.0		1.0	
*C*_max_ (nmol/L)	—	270		162	—	302		161	—	479		301
*T*_max_ (h)	—	0.17		0.5	—	0.17		0.50	—	0.17		0.50
*t*_1/2_ (h)	1.41	—		1.34	1.75	—		1.56	1.49	—		2.74
*AUC*_0→∞_ (nmol/L·h)	886	489		306	1200	564		365	1290	896		601
*CL*_tot_ (L/h/kg)	3.41		—		2.51		—		2.33		—	
*V*d_ss_ (L/kg)	3.55		—		4.36		—		3.70		—	
*k*_a_ (h^−1^)			1.63				1.85				2.18	
*F*_a_ *F*_g_ (%)			46.5				50.6				75.0	
*F*_h_ (%)			74.3				59.9				61.9	
*BA* (%)			34.5				30.3				46.4	

In contrast, *AUC*_sys_ of ciprofloxacin after intravenous injection in Bcrp(−/−) mice was approximately 1.5‐fold higher than that in WT mice (1203 nmol/L·h vs 886 nM·h), and *CL*_tot_ in Bcrp(−/−) mice was lower than that in WT mice (2.51 L/h/kg vs 3.41 L/h/kg). These results suggest that BCRP is involved in the elimination of ciprofloxacin in mice.

On the other hand, *F*_a_ *F*_g_ and *k*_a_ values of ciprofloxacin after oral administration in Mdr1a/1b(−/−) mice were much higher than those in WT mice (75.0% vs 46.5% and 2.18 per hour vs 1.63 per hour, respectively). Moreover, *AUC*_sys_ in Mdr1a/1b(−/−) mice was higher than that in WT mice (601 nmol/L·h vs 306 nmol/L·h), and *CL*_tot_ in Mdr1a/1b(−/−) mice was lower than that in WT mice (2.33 L/h/kg vs 3.41 L/h/kg). These results suggest that p‐gp is involved in both intestinal absorption and elimination process of ciprofloxacin.

#### 3.1.2 Nitrofurantoin

**Figure 4:**
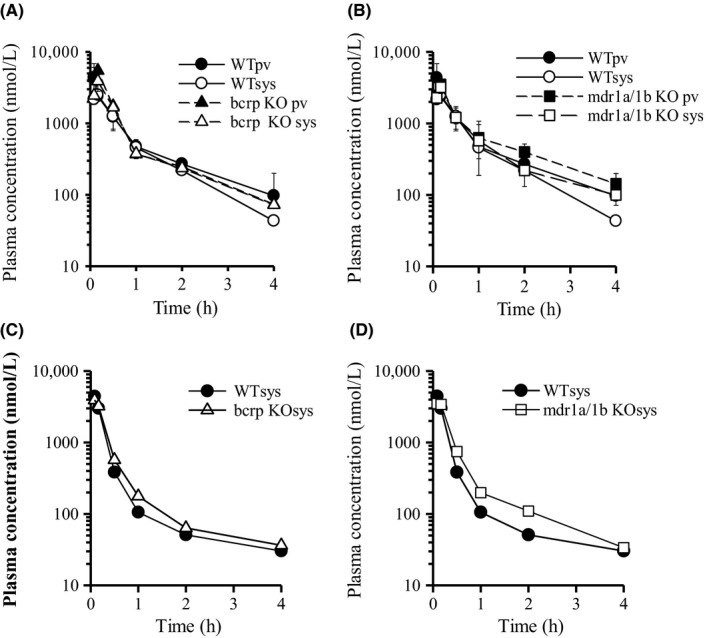
Plasma concentration vs time profiles of nitrofurantoin in WT, Bcrp(−/−), and Mdr1a/1b(−/−) mice after oral and intravenous administration. The plasma concentration vs time profiles of nitrofurantoin in WT, Bcrp(−/−), and Mdr1a/1b(−/−) mice after oral administration (2 mg/kg; A, B) and intravenous administration (1 mg/kg; C, D). Each point is expressed as means ± SD (po: n = 3) or means (iv; n = 2)

The plasma concentration‐time curve of nitrofurantoin after intravenous and oral administration in WT, Bcrp(−/−), and Mdr1a/1b(−/−) mice is shown in Figure 4, and the corresponding pharmacokinetic parameters are listed in Table 2. The *F*_a_ *F*_g_ and *k*_a_ values of nitrofurantoin after oral administration in WT mice were 63.7% and 5.8 per hour, respectively, indicating that nitrofurantoin is well absorbed from the intestine. Moreover, the *F*_a_ *F*_g_ values of nitrofurantoin after oral administration in Bcrp(−/−) mice and Mdr1a/1b(−/−) mice were 77.1% and 75.2%, respectively. The *k*_a_ values in Bcrp(−/−) mice and Mdr1a/1b(−/−) mice were 6.9 per hour and 7.2 per hour, respectively. These *F*_a_ *F*_g_ and *k*_a_ values were higher than those in WT mice. Furthermore, *AUC*_pv_ and *AUC*_sys_ of nitrofurantoin after oral administration in Bcrp(−/−) mice (3414 nmol/L·h and 2557 nmol/L·h) and in Mdr1a/1b(−/−) mice (3182 nmol/L·h and 2345 nmol/L·h) were approximately 1.5‐fold higher than those in WT mice (2545 nmol/L·h and 1835 nmol/L·h). These results suggest that both BCRP and p‐gp are involved in the intestinal absorption of nitrofurantoin.

**Table 2:** Pharmacokinetic parameters of nitrofurantoin after oral and intravenous administration to wild‐type, Bcrp(−/−), and Mdr1a/1b(−/−) mice

	wild‐type				Bcrp(−/−)				Mdr1a/1b(−/−)			
	iv	p.o.			iv	p.o.			iv	p.o.		
		pv		sys		pv		sys		pv		sys
Dose (mg/kg)	1.0		1.0		1.0		1.0		1.0		1.0	
*C*_max_ (nmol/L)	—	3000		2490	—	5480		3910	—	3910		3210
*T*_max_ (h)	—	0.17		0.17	—	0.17		0.17	—	0.17		0.17
*t*_1/2_ (h)	2.87	—		0.93	1.41	—		1.25	1.17	—		1.22
AUC_0→∞_ (nmol/L·h)	1840	2550		1840	1840	3410		2560	1950	3180		2350
CL_tot_ (L/h/kg)	2.28		—		2.28		—		2.15		—	
*V*d_ss_ (L/kg)	1.94		—		1.45		—		1.36		—	
*k*_a_ (h^−1^)			5.80				6.89				7.20	
*F*_a_ *F*_g_ (%)			63.7				77.1				75.2	
F_h_ (%)			78.3				90.0				79.9	
BA (%)			49.9				69.4				60.0	

On the other hand, there were no significant differences in *AUC*_sys_ and *CL*_tot_ values between bcrp knockout (KO) and p‐gp KO mice, indicating that BCRP and p‐gp hardly affect the elimination process of nitrofurantoin.

#### 3.1.3 Topotecan

**Figure 5:**
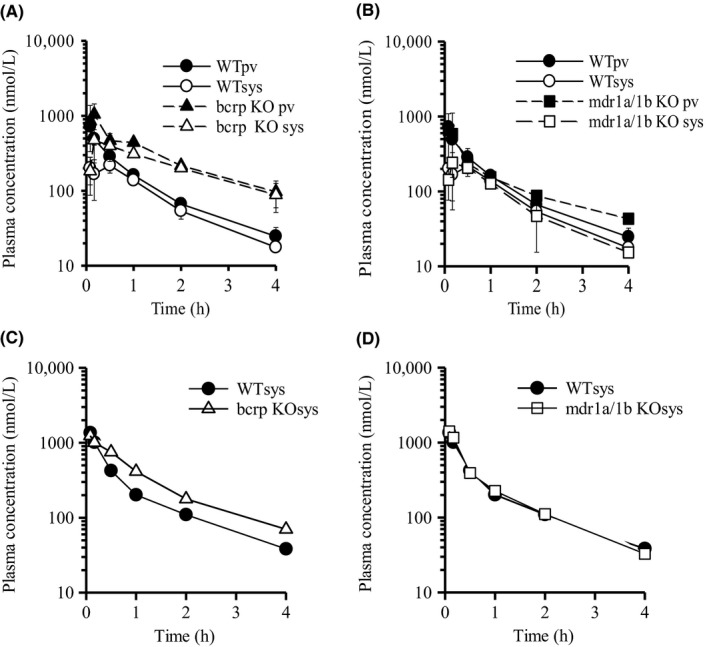
Plasma concentration vs time profiles of topotecan in WT, Bcrp(−/−), and Mdr1a/1b(−/−) mice after oral and intravenous administration. The plasma concentration vs time profiles of topotecan in WT, Bcrp(−/−), and Mdr1a/1b(−/−) mice after oral administration (1 mg/kg; A, B) and intravenous administration (1 mg/kg; C, D). Each point is expressed as means ± SD (po: n = 3) or means (iv; n = 2)

The plasma concentration‐time profiles of topotecan after intravenous and oral administration in WT, Bcrp(−/−), and Mdr1a/1b(−/−) mice were also investigated (Figure 5, Table 3). The *k*_a_ value of topotecan in WT mice was 3.18 per hour, indicating that topotecan is rapidly absorbed from the upper intestine after oral administration. In addition, its BA in WT mice was approximately 37%, and this is similar to the human BA (40%).^15^, ^16^ The *AUC*_pv_ and *AUC*_sys_ values of topotecan after oral administration in Bcrp(−/−) mice were approximately 3‐fold higher than those in WT mice (1509 nmol/L·h vs 577 nmol/L·h and 1136 nmol/L·h vs 370 nmol/L·h, respectively). Moreover, the *F*_a_ *F*_g_ in Bcrp(−/−) mice was 100%, which was much higher than that in WT mice (57%), and its *k_a_* value was 5.18 per hour. These results indicate that the intestinal absorption of topotecan in mice is dominated by BCRP.

**Table 3:** Pharmacokinetic parameters of topotecan after oral and intravenous administration to wild‐type, Bcrp(−/−), and Mdr1a/1b(−/−) mice

	wild‐type				Bcrp(−/−)				Mdr1a/1b(−/−)			
	iv	p.o.			iv	p.o.			iv	p.o.		
		pv		sys		pv		sys		pv		sys
Dose (mg/kg)	1.0		1.0		1.0		1.0		1.0		1.0	
*C*_max_ (nmol/L)	—	720		218	—	855		476	—	664		242
*T*_max_ (h)	—	0.08		0.50	—	0.08		0.17	—	0.08		0.17
*t*_1/2_ (h)	1.26	—		1.04	1.49	—		1.47	1.08	—		1.23
AUC_0→∞_ (nmol/L·h)	994	577		370	1490	1510		1140	1020	589		358
CL_tot_ (L/h/kg)	2.20		—		1.47		—		2.13		—	
*V*d_ss_ (L/kg)	2.59		—		2.21		—		2.19		—	
*k*_a_ (h^−1^)			2.33				5.18				3.68	
*F*_a_ *F*_g_ (%)			56.8				103				63.8	
*F*_h_ (%)			65.6				74.0				54.8	
BA (%)			37.3				76.3				34.9	

The higher level of *AUC*_sys_ and slightly lower *CL*_tot_ value were observed after intravenous injection of topotecan in Bcrp(−/−) mice, compared with WT mice. These results suggest that BCRP is also involved in the elimination process of topotecan.

On the other hand, there were no differences in the pharmacokinetics of topotecan between oral and intravenous administration in Mdr1a/1b(−/−) mice, indicating that p‐gp has no effect on the intestinal absorption and elimination of topotecan.

#### 3.1.4 Sulfasalazine

**Figure 6:**
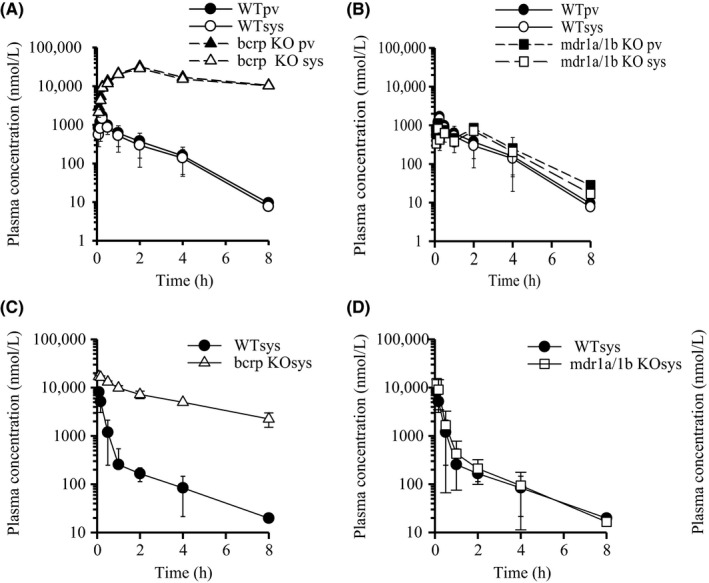
Plasma concentration vs time profiles of sulfasalazine in WT, Bcrp(−/−), and Mdr1a/1b(−/−) mice after oral and intravenous administration. The plasma concentration vs time profiles of sulfasalazine in WT, Bcrp(−/−), and Mdr1a/1b(−/−) mice after oral administration (5 mg/kg; A, B) and intravenous administration (1 mg/kg; C, D). Each point is expressed as means ± SD (po: n = 3) or means (iv; n = 2)

The time course of plasma concentration of sulfasalazine after intravenous and oral administration in WT, Bcrp(−/−), and Mdr1a/1b(−/−) mice is shown in Figure 6, and the corresponding pharmacokinetic parameters are given in Table 4. The *F*_a_ *F*_g_ and BA values in WT mice were estimated to be 16.9% and 10.2%, respectively. These are almost similar to the human *F*_a_ *F*_g_ and BA (12% and < 15%, respectively).^17^, ^18^, ^19^ These results indicate that the intestinal absorption of sulfasalazine is extremely low. In addition, the *t*_1/2_ of sulfasalazine after oral administration in Bcrp(−/−) mice was 7 hours, which was much longer than that in WT mice (1 hour). Moreover, the AUC_pv_ and AUC_sys_ values in Bcrp(−/−) mice were more than 130‐fold higher than those in WT mice (289 955 nmol/L·h vs 2204 nmol/L·h and 287 957 nmol/L·h vs 1943 nmol/L·h, respectively). Furthermore, the *F*_a_ *F*_g_ in Bcrp(−/−) mice was estimated to be about 100%, indicating that BCRP greatly contributes to the intestinal absorption of sulfasalazine.

**Table 4:** Pharmacokinetic parameters of sulfasalazine after oral and intravenous administration to wild‐type, Bcrp(−/−), and Mdr1a/1b(−/−) mice

	wild‐type				Bcrp(−/−)				Mdr1a/1b(−/−)			
	iv	p.o.			iv	p.o.			iv	p.o.		
		pv		sys		pv		sys		pv		sys
Dose (mg/kg)	1.0		1.0		1.0		1.0		1.0		1.0	
*C*_max_ (nmol/L)	—	1180		845	—	32 100		28 800	—	814		629
*T*_max_ (h)	—	0.17		0.50	—	2.00		2.00	—	0.17		0.50
*t*_1/2_ (h)	1.95	—		1.11	3.57	—		7.07	1.63	—		1.11
AUC_0→∞_ (nmol/L·h)	3820	2200		1940	60 500	290 000		288 000	4280	2770		2450
CL_tot_ (L/h/kg)	0.66		—		0.04		—		0.59		—	
*V*d_ss_ (L/kg)	0.59		—		0.19		—		0.45		—	
*k*_a_ (h^−1^)			1.40				0.49				0.93	
*F*_a_ *F*_g_ (%)			16.9				130				20.6	
*F*_h_ (%)			60.1				73.2				55.6	
BA (%)			10.2				95.2				11.5	

However, the *T*_max_ of sulfasalazine after oral administration in Bcrp(−/−) mice was significantly later than that in WT mice (2 hours vs 0.5 hours). In addition, the *CL*_tot_ after intravenous administration in WT mice was 0.66 L/h/kg, whereas that in Bcrp(−/−) mice was 0.04 L/h/kg. These results indicate that BCRP also strongly influences the elimination of sulfasalazine. Interestingly, extrapolated plasma concentration at zero time (*C*_0_) after intravenous injection of sulfasalazine in Bcrp(−/−) mice was higher than that in WT mice (18.1 μmol/L vs 11.7 μmol/L). In addition, the distribution phase in Bcrp(−/−) mice was hardly observed, and the *V*_dss_ was low (0.19 L/kg). Taken together, it is considered that the late *t*_max_ and small *V*_dss_ values cause the low *k*_a_ value of sulfasalazine despite its *F*_a_ *F*_g_ value was approximately 100%.

On the other hand, the *F*_a_ *F*_g_ value of sulfasalazine in Mdr1a/1b(−/−) mice (*F*_a_ *F*_g_: 30%) was also higher than that in WT mice. However, the influence of p‐gp on the intestinal absorption of sulfasalazine is considered not to be so high compared with BCRP. In addition, the *C*_0_ and *V*_dss_ values were not different between Mdr1a/1b(−/−) mice and WT mice.

Sulfasalazine is degraded to sulfapyridine and 5‐aminosalicylic acid by bacteria in the large intestine.^17^ Although sulfapyridine is well absorbed from the intestine, its plasma concentration in Bcrp(−/−) mice was much less than that in WT and Mdr1a/1b(−/−) mice (data not shown). This may be because sulfasalazine is highly absorbed from the intestine without degradation in Bcrp(−/−) mice, whereas sulfasalazine is degraded to sulfapyridine because of its low *F*_a_ *F*_g_ in WT and Mdr1a/1b(−/−) mice.

We summarized the *k*_a_ values and calculated AQ_bcrp_ and AQ_P‐gp_ values of model drugs in Table 5. The *k*_a_ values of model drugs, except sulfasalazine, were higher in Bcrp(−/−) and Mdr1a/1b(−/−) mice than in WT mice, suggesting that these transporters affect their intestinal absorption. However, each model drug showed different AQ value, implying that the degree of the contribution of these transporters differs among them. Although topotecan showed slightly higher AQ_bcrp_ value of 0.35, other drugs showed low AQ_bcrp_ and AQ_P‐gp_ values. This suggests that both BCRP and P‐gp hardly affect the intestinal absorption of ciprofloxacin and nitrofurantoin in mice. In sulfasalazine, AQ value could not be estimated because the *k_a_* value was much lower in Bcrp(−/−) mice despite its *F*_a_ *F*_g_ value was significantly higher than WT mice.

**Table 5:** Data summary for *k*_a_ and in vivo AQ values

Compound	*k*_a, wt_	*k*_a, bcrp_	*k*_a, P‐gp_	*AQ*_bcrp_	*AQ*_P‐gp_
ciprofloxacin	1.63	1.85	2.18	0.09	0.19
nitrofurantoin	5.80	6.89	7.20	0.13	0.14
topotecan	3.18	5.18	3.68	0.35	0.08
sulfasalazine	1.40	0.49	0.93	—	—

Then, we calculated the rate of contribution (*R*) value on the intestinal absorption using *F*_a_ *F*_g_ values in each mice (Table 6). Ciprofloxacin and nitrofurantoin showed low *R*_bcrp_ and *R*_p‐gp_ values, indicating that the contribution of both BCRP and P‐gp to their intestinal absorption would be little. On the other hand, topotecan and sulfasalazine showed relatively high *R*_bcrp_ in contrast to low *R*_p‐gp_. These results indicate that BCRP mainly acts as a barrier to their intestinal absorption.

**Table 6:** Data summary for *F*_a_ *F*_g_ and rate of contribution (*R*) values in vivo

Compound	(*F*_a_ *F*_g_) _wt_	(*F*_a_ *F*_g_)_bcrp_	(*F*_a_ *F*_g_)_P‐gp_	*R*_bcrp_	*R*_P‐gp_
ciprofloxacin	0.47	0.51	0.75	0.05	0.36
nitrofurantoin	0.64	0.77	0.75	0.15	0.13
topotecan	0.57	1.03	0.64	0.42	0.06
sulfasalazine	0.16	1.30	0.30	0.79	0.09

### 3.2 Evaluation of the in vitro‐in vivo correlation

**Figure 7:**
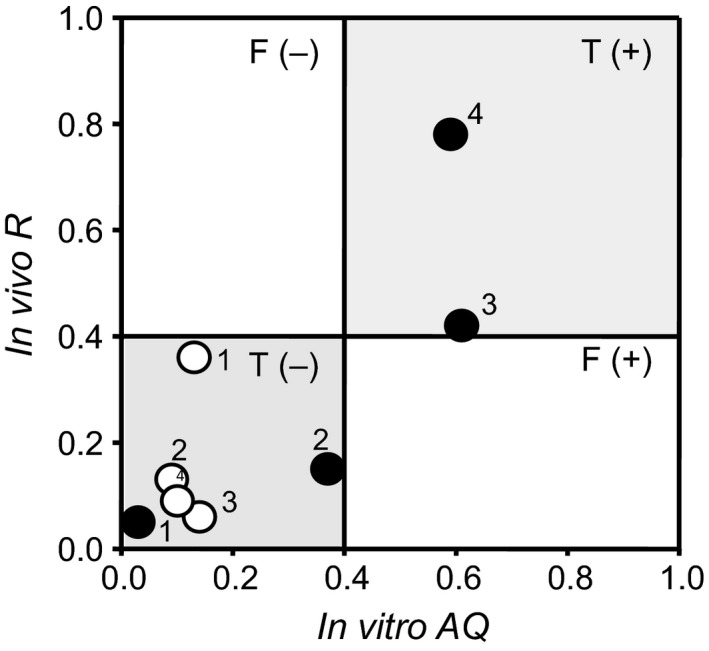
Relationship between in vitro *AQ* and in vivo contribution ratio (*R*) for 4 model drugs. Open or closed circles represent the relationship between in vitro AQ_P‐gp_ and in vivo *R*_P‐gp_ or in vitro AQ_BCRP_ and in vivo *R*_bcrp_, respectively. In vitro AQ values were cited from our previous report (Kawahara et al, manuscript in revision). Key: 1, ciprofloxacin; 2, nitrofurantoin; 3, topotecan; 4, sulfasalazine. F (˗): False negative, F (+): False positive, T (˗): True negative, T (+): True positive

We have demonstrated that the *R* value would be a valuable alternative parameter to in vivo AQ for estimating the contribution of efflux transporters to drug absorption. Therefore, we investigated the relationship between in vivo *R* and in vitro AQ estimated from Caco‐2 permeability in our previous study.We have clarified that the drugs, which show the AQ value of more than 0.4, tend to be limited their intestinal permeability by P‐gp (Fujita et al, manuscript in preparation). In addition, our previous report has demonstrated that BCRP highly contributes to the transport of the model compounds with the *R* value of above 0.4 in Caco‐2 cell monolayer.Moreover, the present study suggests that BCRP acts as an absorptive barrier to the drugs which have the *R* value above 0.4. Based on these findings, we set criteria of AQ and *R* at 0.4 for the risk of efflux transporters for limiting the intestinal absorption of drugs. The drugs used in this study were categorized in four classes according to in vitro AQ and in vivo *R* values (Figure 7). All the drugs belonged to the upper right or lower left class. These findings suggest that the AQ value assessed from in vitro Caco‐2 permeability study is useful for the accurate estimation of the contribution of BCRP and p‐gp to in vivo intestinal absorption.

## 4 DISCUSSION

In this study, we defined the *R* value for estimating the quantitative contribution of BCRP and P‐gp to the intestinal absorption of drugs, by P‐S difference method in WT, Bcrp(−/−), and Mdr1a/1b(−/−) mice. In addition, we showed the close relationship between in vivo *R* value and in vitro AQ value. Moriwaki et al have determined the pharmacokinetic parameters, including *AUC*, *F*_a_ *F*_g_, and BA, of several drugs by P‐S difference method in rats, and they have demonstrated that these parameters can be more strictly defined than those by the simplified models.^20^, ^21^ In addition, the pharmacokinetic parameters of drugs evaluated by P‐S difference method were in good accordance with the experimental values obtained from other recirculatory models, such as bile duct cannulation method. Moreover, P‐S difference method can define the drug pharmacokinetics on a physiological basis without significant experimental variability. Based on these reasons, we used P‐S difference method here to determine the local drug absorption.

In ciprofloxacin, P‐gp, not BCRP, was likely to mainly contribute to its intestinal absorption (Figure 3, Tables 1 and 5). However, its *F*_a_ *F*_g_ value was about 0.5 in WT mice, and it has been reported that the oral BA of ciprofloxacin in human is about 70%.^22^ Therefore, the effect of P‐gp would not become an important issue in the intestinal absorption of ciprofloxacin. In addition, the present results suggest the involvement of BCRP and P‐gp in the elimination process of ciprofloxacin. Ando et al have reported that the biliary excretion clearance and kidney/plasma concentration ratio of ciprofloxacin are about 3‐fold higher in Bcrp(−/−) mice than in WT mice, indicating the contribution of BCRP to both the biliary excretion and tubular secretion.^23^ However, the main elimination pathway of ciprofloxacin is urinary excretion in human, and BCRP has been reported not to be expressed in human kidney.^24^ Therefore, it is conceivable that the drug‐drug interaction in BCRP is unlikely to occur through the elimination process.

Then, it is suggested that both BCRP and P‐gp affect the intestinal absorption of nitrofurantoin in mice (Figure 4, Tables 2 and 5). However, their contribution to the intestinal absorption could be ignored because nitrofurantoin showed high *F*_a_ *F*_g_ value in WT mice and there are no clinical reports about the involvement of P‐gp in its absorption.

On the other hand, the intestinal absorption of topotecan was highly affected by BCRP (Figure 5, Tables 3 and 5), although it has been reported that the distribution of topotecan is restricted by P‐gp, rather than BCRP, in brain.^25^ Moreover, the possibility of the involvement of BCRP in the elimination process was also demonstrated in the present study. It has been reported that the urinary excretion is the main elimination pathway of topotecan in mice and human.^26^, ^27^ On the other hand, Jonker et al have shown that GF120918, a BCRP inhibitor, decreases the biliary excretion of topotecan after intravenous administration, while its urinary excretion is hardly affected by GF120918.^28^ Taking these findings into consideration, it is conceivable that the involvement of BCRP in biliary excretion of topotecan results in its lower *CL*_tot_ in Bcrp(−/−) KO mice. In human clinical studies, it has been demonstrated that topotecan shows the poor BA after oral administration (about 40%), and it is hardly metabolized.^16^ However, the BA of topotecan has been reported to significantly increase to 100% when GF120918 is orally coadministered.^30^, ^31^ Furthermore, Sparreboom et al have shown that the oral BA of topotecan is 1.3‐fold higher in patients who are heterozygous variant for the BCRP single‐nucleotide polymorphism (SNP) than in patients with the normal BCRP.^31^ These results are in accordance with our present results. Taken together, BCRP would act as a barrier for oral absorption of topotecan in human.

Similar to topotecan, the intestinal absorption of sulfasalazine was highly influenced by BCRP, rather than P‐gp (Figure 6, Table 3 and 5). Interestingly, the smaller *V*_dss_ was observed in Bcrp(−/−) mice than WT mice, despite the *V*_dss_ is assumed to become higher in Bcrp(−/−) mice than WT mice because BCRP is expressed in various tissues. These results are in accordance with the previous findings reported by Karibe et al and Liao et al.^32^, ^33^ Since Zaher et al have confirmed that the plasma protein binding of sulfasalazine is the same in both WT and Bcrp(−/−) mice,^34^ the protein binding would not be related to the small *V*_dss_ value in Bcrp(−/−) mice. Similar phenomenon has also been reported in other compounds,^8^ and further investigation is required to clarify this event. In human study, the *AUC* value of sulfasalazine after oral administration in patients who are heterozygous variant for the *BCRP* SNP has been reported to be approximately 2‐fold higher than that in patients with the normal *BCRP*.^35^ Since there are no differences in the elimination of sulfasalazine between those patients, BCRP would affect the oral absorption of sulfasalazine not only in mice but also in human.

Thus, we have revealed that in vivo pharmacokinetic parameters of topotecan and sulfasalazine, which showed relatively high *R*_bcrp_ value, could well reflect the human situation. Therefore, we finally compared in vivo *R*_bcrp_ values with in vitro AQ values obtained from Caco‐2 permeability studies (Figure 7, Table 7). Ciprofloxacin and nitrofurantoin, which showed low *R*_bcrp_ value in vivo, showed low AQ_BCRP_ values in vitro. In contrast, topotecan and sulfasalazine, which have been shown to be greatly influenced their oral absorption by BCRP in human, showed both in vivo *R*_bcrp_ and in vitro AQ_BCRP_ of more than 0.4. However, their absolute values were different in each drug, suggesting that the substrate recognition property of BCRP differ between mice and human. On the other hand, in vitro AQ_BCRP_ value of topotecan was comparable to human AQ_BCRP_ value estimated from the clinical data (0.61 vs 0.58).^30^, ^31^

**Table 7:** Comparison between in vitro AQ and in vivo rate of contribution (*R*) for BCRP and P‐gp

Compound	in vitro (Caco‐2)		in vivo (mice)	
	AQ_bcrp_ a	AQ_p‐gp_ b	*R*_bcrp_	*R*_P‐gp_
ciprofloxacin	0.03	0.13	0.05	0.36
nitrofurantoin	0.37	0.09	0.15	0.13
topotecan	0.61	0.14	0.42	0.06
sulfasalazine	0.59	0.05	0.79	0.09

a In vitro *AQ*_bcrp_ values are cited from (10).

b Unpublished data (Fujita et al, manuscript in preparation).

In conclusion, we demonstrate that the accurate prediction of the contribution of BCRP in human intestinal drug absorption could be achieved using in vitro *AQ*_BCRP_ calculated from Caco‐2 permeability studies. Further investigation using other BCRP substrates with various affinity is needed to demonstrate the validity of our prediction. Nevertheless, our present observations make a valuable contribution toward the construction of database for the precise prediction of human intestinal drug absorption.

## DISCLOSURE

The authors declare that the research was conducted in the absence of any commercial or financial relationships that could be construed as a potential conflict of interest. This work did not involve studies with human subjects.

## AUTHOR CONTRIBUTIONS

IK, AY, and TF have made substantial contributions to conception and design of the work. IK, SN, YK, and TF have made substantial contributions to the acquisition of data, their analysis, and their interpretation. IK, YK, SN, and TF have been involved in drafting the manuscript. YK, IK, and TF revised the manuscript critically for important intellectual content. All authors have given final approval of the version to be published.

## ETHIC STATEMENT

All animal experimental protocols were reviewed and approved by the Animal Care and Use Committee of Kyoto Pharmaceutical University (2005‐239) and Ritsumeikan University (BKC2010‐27).

## Data Availability

The data that support the findings of this study are available from the corresponding author upon reasonable request.
